# Early Computed Tomography Imaging and Clinical Outcomes in Hospitalized Patients with Acute Diverticulitis: A Propensity-Matched Multicenter Cohort Study

**DOI:** 10.3390/diagnostics16152322

**Published:** 2026-07-24

**Authors:** Noor Albusta, Ali Bosta, Hussain Alrahma

**Affiliations:** 1 Department of Internal Medicine, Lahey Hospital and Medical Center, Burlington, MA 01805, USA; 2 Medical Department, Salmaniya Medical Complex, Manama P.O. Box 12, Bahrain; aalbosta@hospitals.gov.bh; 3 Department of Gastroenterology and Hepatology, Salmaniya Medical Complex, Manama P.O. Box 12, Bahrain; hrahma1@hospitals.gov.bh

**Keywords:** diverticulitis, computed tomography, diagnostic imaging, older adults, hospitalization, mortality, bowel surgery, sepsis, acute kidney injury, propensity score matching

## Abstract

**Background/Objectives:** Computed tomography (CT) is the gold-standard diagnostic modality for acute diverticulitis, yet the association between early CT imaging and short-term clinical outcomes among hospitalized older adults has not been evaluated in a large multicenter propensity-matched cohort. We assessed whether early CT abdomen/pelvis (within 24 h of admission) was associated with improved short-term outcomes compared with delayed or absent CT among adults aged ≥65 years hospitalized with acute diverticulitis. **Methods:** We conducted a retrospective cohort study using the TriNetX US Collaborative Research Network through February 2026. Early CT was defined as CT abdomen/pelvis performed within 24 h of the index hospitalization. Patients underwent 1:1 propensity score matching using demographic, comorbidity, diverticulitis-related, medication, and laboratory variables. The primary outcome was 30-day all-cause mortality. **Results:** Among 86,214 eligible patients, 62,478 received early CT and 23,736 did not. After matching, 22,840 patients remained in each cohort. Early CT was associated with lower 30-day mortality (RR 0.72, 95% CI 0.62–0.84), 90-day mortality (RR 0.78, 95% CI 0.70–0.87), bowel surgery within 30 days (RR 0.81, 95% CI 0.73–0.90), sepsis (RR 0.76, 95% CI 0.69–0.84), acute kidney injury (RR 0.85, 95% CI 0.78–0.92), ICU admission (RR 0.79, 95% CI 0.72–0.87), and 30-day readmission (RR 0.86, 95% CI 0.80–0.93). Early CT was also associated with higher rates of percutaneous abscess drainage (RR 1.37, 95% CI 1.21–1.55) and shorter hospital length of stay (5.4 vs. 6.8 days; mean difference −1.4 days, 95% CI −1.6 to −1.2). **Conclusions:** Early CT imaging within 24 h of admission was associated with improved short-term outcomes among older adults hospitalized with acute diverticulitis, including lower mortality, reduced need for bowel surgery, lower rates of sepsis and acute kidney injury, shorter hospital stay, and higher utilization of percutaneous abscess drainage. These findings support guideline recommendations for prompt diagnostic CT imaging in this population and suggest that early CT may facilitate timely risk stratification, source control, and management optimization.

## 1. Introduction

Diverticulitis is defined as inflammation of colonic diverticula and is one of the most common gastrointestinal conditions requiring hospitalization in the United States, accounting for approximately 200,000 hospital admissions annually with an estimated healthcare expenditure exceeding $6.3 billion per year [1,2]. The annual incidence of diverticulitis in the US is approximately 180–188 per 100,000 persons, and the incidence increases with age [1,2,3,4]. Risk factors for diverticular disease include age older than 65 years, body mass index ≥30, use of opioids, corticosteroids, and nonsteroidal anti-inflammatory medications, hypertension, and type 2 diabetes [1,2].

Approximately 85% of patients with acute diverticulitis have uncomplicated disease, while 12–15% present with complicated diverticulitis, including phlegmon, abscess, perforation, obstruction, stricture, or fistula [1,2,3,4,5]. Among older adults, diverticulitis presents unique diagnostic and management challenges. Older patients are less likely to have typical signs and symptoms: patients older than 80 years have lower rates of fever (21.4% vs. 35.2%), abdominal pain (47.8% vs. 65.6%), and leukocytosis compared with younger patients [3,4]. Clinical suspicion alone is correct in only 40–65% of patients, underscoring the critical role of diagnostic imaging [6,7,8].

**Figure 1:**
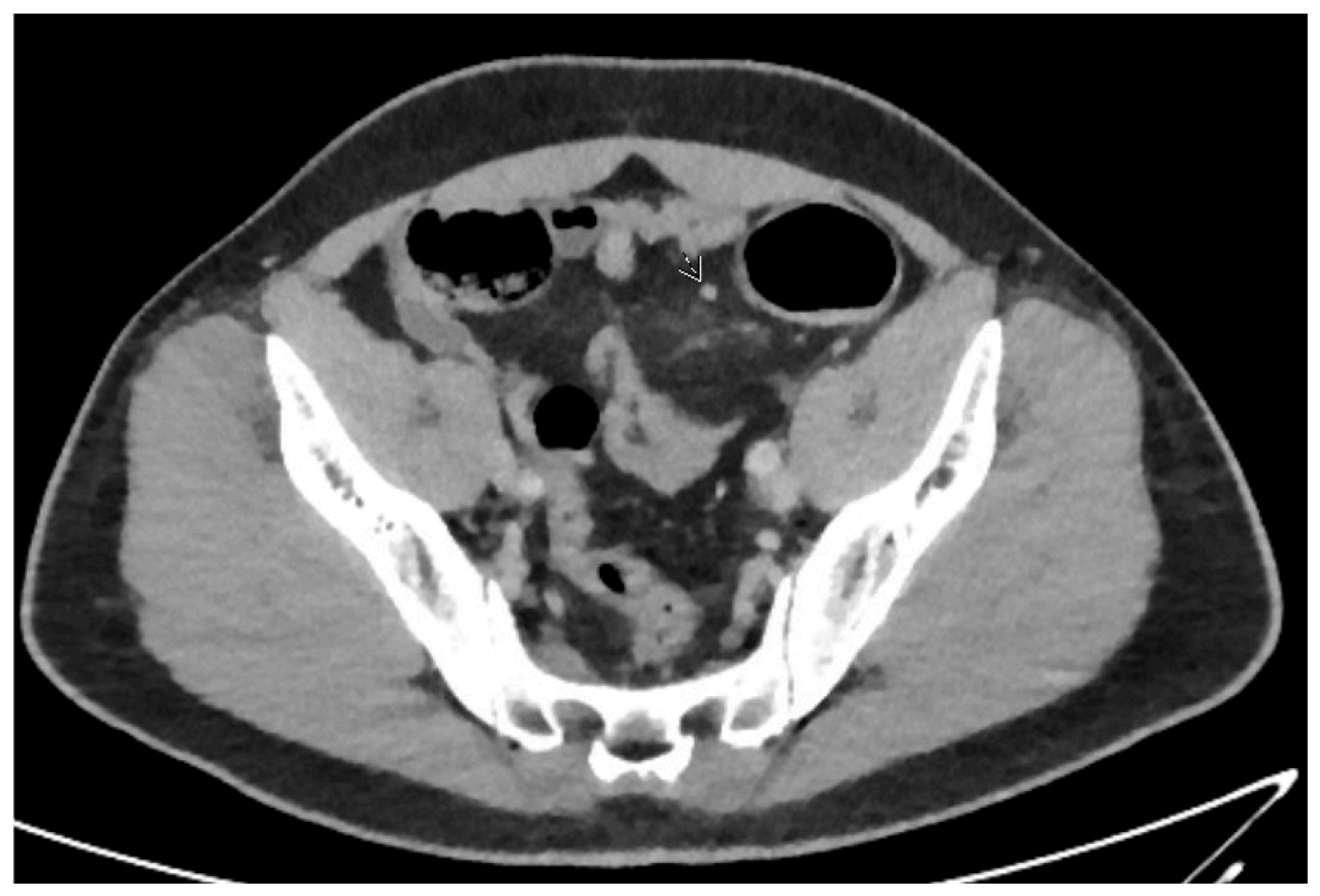
Axial contrast-enhanced CT of the pelvis demonstrating focal sigmoid colonic wall thickening with surrounding pericolic inflammatory stranding and a small adjacent gas-containing focus, compatible with acute diverticulitis.

**Figure 2:**
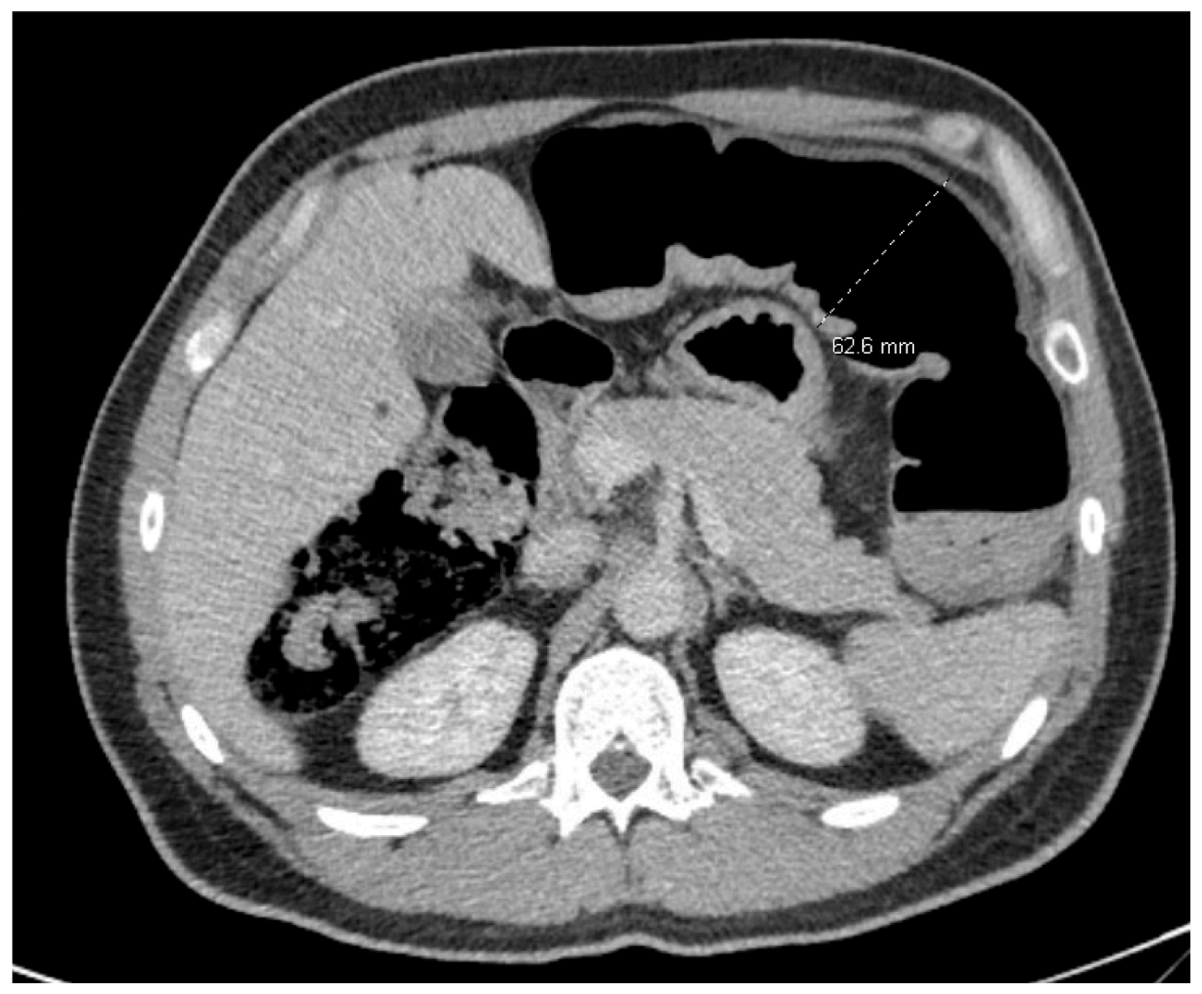
Axial contrast-enhanced CT of the abdomen demonstrating marked proximal colonic dilatation, measuring approximately 6.3 cm, secondary to a diverticulitis-related colonic stricture causing mechanical large-bowel obstruction.

Computed tomography (CT) of the abdomen and pelvis with intravenous contrast is the recommended diagnostic test for acute diverticulitis, with a sensitivity of 98–99% and specificity of 99–100% [1,2,5]. The American Society of Colon and Rectal Surgeons (ASCRS) provides a strong recommendation (Grade 1B) that CT is the most appropriate initial imaging modality in the assessment of suspected diverticulitis [9]. The American College of Radiology (ACR) Appropriateness Criteria similarly designate CT as the most useful examination for suspected colonic diverticulitis [6]. Representative CT manifestations of diverticular disease are shown in Figure 1 and Figure 2. Figure 1 demonstrates acute sigmoid diverticulitis with focal mural thickening, surrounding pericolic inflammatory change, and a small adjacent gas-containing focus.

Figure 2 demonstrates a diverticulitis-related colonic stricture causing mechanical large-bowel obstruction with marked proximal colonic dilatation.

These examples illustrate how CT can identify both active inflammation and clinically important complications that may alter management. The American Gastroenterological Association (AGA) recommends CT to confirm the diagnosis in patients without prior imaging-confirmed disease and to evaluate for complications in severe presentations [7]. The American College of Physicians (ACP) suggests CT imaging when diagnostic uncertainty exists [8].

Beyond diagnostic confirmation, CT imaging serves several critical functions in acute diverticulitis management: grading disease severity according to the modified Hinchey classification, identifying complications requiring intervention (abscess amenable to percutaneous drainage, perforation requiring surgery), risk-stratifying patients for operative versus nonoperative management, and facilitating inpatient versus outpatient triage [5,6,9]. CT can predict unfavorable outcomes from acute diverticulitis, as longer segments of involved colon, retroperitoneal abscess, and extraluminal air have been associated with recurrence, failure of medical management, and need for surgery [6]. Early CT diagnosis of uncomplicated diverticulitis in the emergency department has been shown to reduce hospital admission by more than 50% and shorten hospital length of stay [6].

Despite strong guideline support for CT imaging in acute diverticulitis, the specific association between early CT imaging (within 24 h of admission) and short-term clinical outcomes among hospitalized older adults has not been evaluated in a large multicenter propensity-matched cohort. Older adults are particularly relevant because they present with atypical symptoms, have higher rates of complicated disease, and experience substantially higher postoperative mortality (9.7% in patients aged 65–79 years and 17.8% in patients ≥80 years, compared with 1.6% in patients <65 years) [2,3,4]. Guidelines support a lower threshold for obtaining diagnostic imaging in this population [3,4,8].

To address this gap, we conducted a retrospective cohort study using the TriNetX US Collaborative Research Network. We evaluated whether early CT abdomen/pelvis within 24 h of admission was associated with mortality, bowel surgery, percutaneous abscess drainage, sepsis, acute kidney injury, intensive care unit admission, venous thromboembolism, Clostridioides difficile infection, readmission, and hospital length of stay after propensity score matching for demographic, comorbidity, diverticulitis-related, medication, and laboratory variables.

## 2. Materials and Methods

### 2.1. Study Design

We performed a retrospective cohort study using the TriNetX US Collaborative Research Network (TriNetX LLC, Cambridge, MA, USA), a federated electronic health record database that aggregates de-identified data from healthcare organizations across the United States. Available data include demographics, diagnoses, procedures, medications, laboratory values, vital signs, and healthcare encounters. Cohort identification, data extraction, propensity score matching, and statistical analyses were conducted using the TriNetX Analytics Platform (version 1.0, TriNetX LLC, Cambridge, MA, USA). This study used de-identified, HIPAA-compliant data and therefore did not require institutional review board approval. The study was conducted in accordance with the STROBE guidelines for observational research [10].

Because TriNetX is a federated EHR network, patient-level chart review, radiologist interpretation details, CT protocol specifications, exact imaging-to-report turnaround times, modified Hinchey classification, operative details, and physician global assessment are not uniformly available. Therefore, exposures and outcomes were defined using structured diagnosis, procedure, medication, laboratory, and encounter data. CT contrast protocol could be determined only when the relevant CPT or ICD-10-PCS code explicitly identified contrast administration. Radiology report text, CT image-level findings, abscess dimensions, the anatomical distribution of extraluminal air or free fluid, and detailed operative reports were not uniformly available. Consequently, radiological severity could not be classified using the modified Hinchey or WSES systems.

### 2.2. Study Population and Cohort Definitions

We identified adults aged ≥65 years hospitalized with acute diverticulitis through February 2026. Acute diverticulitis was identified using ICD-10-CM codes K57.20 (diverticulitis of large intestine with perforation and abscess without bleeding), K57.21 (diverticulitis of large intestine with perforation and abscess with bleeding), K57.32 (diverticulitis of large intestine without perforation or abscess without bleeding), K57.33 (diverticulitis of large intestine without perforation or abscess with bleeding), K57.40 (diverticulitis of both small and large intestine with perforation and abscess without bleeding), K57.52 (diverticulitis of both small and large intestine without perforation or abscess without bleeding), K57.80 (diverticulitis of intestine, part unspecified, with perforation and abscess without bleeding), and K57.92 (diverticulitis of intestine, part unspecified, without perforation or abscess without bleeding). The index date was defined as the date of the qualifying inpatient encounter.

To improve specificity for acute diverticulitis-related hospitalization, the primary cohort required a diverticulitis ICD-10-CM code to be recorded as the primary diagnosis or encounter-associated diagnosis for the index hospitalization when available in TriNetX. In addition, patients were required to have at least one acute presentation feature recorded during the index hospitalization, including abdominal pain diagnosis, fever, elevated white blood cell count, elevated C-reactive protein, intravenous antibiotic administration, surgical consultation, or abdominal imaging. This operational definition was intended to enrich for acute diverticulitis admissions while acknowledging that administrative data cannot perfectly distinguish acute diverticulitis-driven hospitalizations from admissions in which diverticulitis is a comorbidity diagnosis.

Patients were classified into two cohorts according to the timing of CT abdomen/pelvis imaging relative to the index hospitalization.

Early CT cohort: Patients who received CT abdomen/pelvis within 24 h of the index hospitalization date were assigned to this group. CT abdomen/pelvis was identified using CPT code 74176 for CT abdomen and pelvis without contrast, CPT code 74177 for CT abdomen and pelvis with intravenous contrast, and CPT code 74178 for CT abdomen and pelvis without contrast followed by imaging with contrast, as well as corresponding ICD-10-PCS imaging codes when available.

Delayed/no CT cohort: Patients hospitalized with acute diverticulitis who did not receive CT abdomen/pelvis within 24 h of admission were included in this cohort. For descriptive purposes, this cohort was further divided into patients who underwent CT more than 24 h after admission but before discharge and patients with no recorded CT abdomen/pelvis during the index hospitalization. The prespecified primary analysis retained these patients within a single comparator cohort according to the absence of CT within the first 24 h.

Exclusion criteria applied to both cohorts included age <65 years, incomplete demographic data, colorectal malignancy before index hospitalization (to exclude patients in whom diverticulitis may be a secondary finding during cancer workup), elective colonic surgery without acute diverticulitis-related hospitalization, prior total colectomy, and insufficient baseline data for propensity matching. Patients missing age, sex, or race/ethnicity were excluded. Patients were not excluded solely for missing laboratory or BMI values; missingness handling is described below.

### 2.3. Baseline Characteristics and Covariates

Baseline variables were selected a priori based on clinical relevance and availability in TriNetX. Demographic variables included age, sex, race/ethnicity, and body mass index when available. Comorbidities documented within 12 months before index hospitalization included hypertension, type 2 diabetes, chronic kidney disease, coronary artery disease, heart failure, chronic obstructive pulmonary disease, dementia, depression, chronic liver disease, and a history of venous thromboembolism.

Diverticulitis-related variables included prior diverticulitis episodes, prior diverticular abscess, prior colonic surgery, complicated diverticulitis coding at index admission (perforation/abscess codes K57.20, K57.21, K57.40, K57.80), prior hospitalization within 12 months, anticoagulant exposure, antiplatelet exposure, corticosteroid exposure, immunosuppressant exposure, opioid exposure, and nonsteroidal anti-inflammatory drug exposure. Laboratory covariates measured within 72 h before or during the index hospitalization included hemoglobin, white blood cell count, platelet count, creatinine, albumin, C-reactive protein, and lactate when available.

Among the 86,214 eligible patients, BMI data were available for 64,660 (75.0%), hemoglobin for 79,317 (92.0%), white blood cell count for 80,180 (93.0%), creatinine for 80,611 (93.5%), albumin for 58,184 (67.5%), platelet count for 78,454 (91.0%), C-reactive protein for 42,486 (49.3%), and lactate for 38,796 (45.0%). Structured complicated-diverticulitis codes indicating perforation or abscess were used as an administrative proxy for greater disease severity. These codes were not considered equivalent to modified Hinchey or WSES classification because they do not reliably distinguish localized pericolic inflammation or air, distant intraperitoneal or retroperitoneal air, diffuse free fluid, purulent peritonitis, or feculent peritonitis. Missing data proportions were similar between the early CT and delayed/no CT cohorts for most variables, although CRP and lactate missingness were modestly higher in the delayed/no CT cohort (CRP: 53.8% vs. 48.6%; lactate: 58.2% vs. 53.4%). Missing-indicator terms were included in the propensity score model for all covariates with incomplete data, as described below.

### 2.4. Study Endpoints and Outcome Definitions

The primary outcome was 30-day all-cause mortality after index hospitalization.

Secondary outcomes included 7-day and 90-day all-cause mortality; bowel surgery within 30 days, including colonic resection, sigmoidectomy, Hartmann procedure, colostomy creation, or other intestinal resection; percutaneous abscess drainage within 30 days; sepsis within 30 days; acute kidney injury within 30 days; intensive care unit admission within 30 days; venous thromboembolism within 30 days; Clostridioides difficile infection within 30 days; blood transfusion within 30 days; 30-day and 90-day all-cause readmission; and hospital length of stay. Percutaneous abscess drainage was evaluated separately as the principal non-resectional source-control intervention that could be reliably identified from structured procedure codes. Other non-resectional operative interventions, including laparoscopic lavage, operative washout, primary closure of a perforation, and combined procedures, could not be reliably classified. These interventions may be represented by overlapping or nonspecific procedure codes, and operative-report text was not available for confirmation. They were therefore not reported as separate outcomes.

Outcomes were defined using ICD-10-CM diagnosis codes, ICD-10-PCS procedure codes, CPT codes, medication records, and encounter data available within TriNetX. Sepsis was identified using ICD-10-CM A40.x, A41.x, R65.2x, and related severe sepsis or septic shock codes. Acute kidney injury was identified using ICD-10-CM N17.x. Sepsis and acute kidney injury were identified as recorded clinical diagnoses using structured ICD-10-CM codes and were not independently adjudicated through patient-level chart review. Laboratory values were used as baseline covariates and selected continuous secondary outcomes but were not used to replace the prespecified coded outcome definitions. A laboratory-only definition of sepsis was not applied because sepsis requires infection-associated organ dysfunction and cannot be reliably identified from isolated laboratory thresholds. A complete KDIGO definition of acute kidney injury could not be uniformly applied because urine-output measurements and sufficiently frequent serial creatinine values were not consistently available across participating healthcare organizations. Venous thromboembolism was identified using ICD-10-CM I26.x, I80.x, I82.x, and related pulmonary embolism or deep venous thrombosis codes. Clostridioides difficile infection was identified using ICD-10-CM A04.71, A04.72. Bowel surgery was identified using ICD-10-PCS and CPT procedure codes for colonic resection, sigmoidectomy, Hartmann procedure, colostomy creation, and related intestinal resection procedures. Percutaneous abscess drainage was identified using CPT codes for image-guided percutaneous drainage (49405, 49406, 49407) and corresponding ICD-10-PCS drainage codes. Blood transfusion was identified using ICD-10-PCS transfusion codes, CPT transfusion codes, or blood product administration records. Intensive care unit admission was identified using ICU encounter/location data when available, or critical care encounter codes such as CPT 99291 and 99292 when location-based data were unavailable. Readmission was defined as a subsequent inpatient encounter within 30 or 90 days after the index discharge or index encounter date. Mortality was identified using the TriNetX structured mortality status within the specified follow-up window.

### 2.5. Statistical Analysis

Baseline characteristics were compared using chi-square tests for categorical variables and Student’s *t*-tests or Wilcoxon rank-sum tests for continuous variables, as appropriate. We performed 1:1 propensity score matching using nearest-neighbor matching without replacement. A caliper width of 0.2 standard deviations of the logit of the propensity score was used, consistent with conventional recommendations for propensity score matching in observational studies.

Covariates included demographics, comorbidities, diverticulitis-related variables (prior episodes, prior abscess, prior colonic surgery, complicated diverticulitis coding, prior hospitalization), medication exposures (anticoagulants, antiplatelets, corticosteroids, immunosuppressants, opioids, NSAIDs), laboratory parameters, and missing-indicator terms for covariates with incomplete data. Covariate balance after matching was assessed using standardized mean differences (SMDs), with values <0.10 considered balanced. The C-statistic of the propensity score model was recorded as a measure of discrimination.

For binary outcomes, relative risks (RRs) and risk differences (RDs) with 95% confidence intervals (CIs) were calculated. Continuous outcomes were reported as means with standard deviations and compared using mean differences with 95% CIs. Cox proportional hazards models were performed in the matched cohort for time-to-event outcomes, and hazard ratios (HRs) with 95% CIs were reported. The proportional hazards assumption was assessed using Schoenfeld residuals for all Cox models reported. No statistically significant violations of the proportional hazards assumption were identified for any of these models (all global test *p* > 0.05).

Sensitivity analyses included (1) restriction to patients with complicated diverticulitis coding (perforation/abscess codes) to assess whether early CT associations persisted in higher-acuity presentations; (2) exclusion of patients who received CT within 6 h (to assess whether very early CT was driving the associations, potentially reflecting confounding by indication in the most acutely ill patients); (3) complete-case analysis among patients with available key laboratory covariates; and (4) subgroup analyses stratified by complicated versus uncomplicated diverticulitis coding, prior diverticulitis episodes, and immunosuppressant exposure. Subgroup analyses were exploratory, and interaction testing was used to assess heterogeneity.

To address heterogeneity within the delayed/no CT comparator cohort, we performed an exploratory three-group analysis comparing patients who underwent early CT within 24 h of admission, delayed CT more than 24 h after admission but before discharge, and no recorded CT abdomen/pelvis during the index hospitalization. This analysis was conducted in the full eligible cohort, with adjustment for the same demographic, comorbidity, diverticulitis-related, medication, laboratory, and missing-indicator covariates included in the primary propensity score model. Adjusted relative risks and 95% confidence intervals were estimated using modified Poisson regression with robust standard errors.

Statistical significance was defined as a two-sided *p*-value < 0.05. All analyses were conducted within the TriNetX analytics platform.

## 3. Results

### 3.1. Study Population

**Figure 3:**
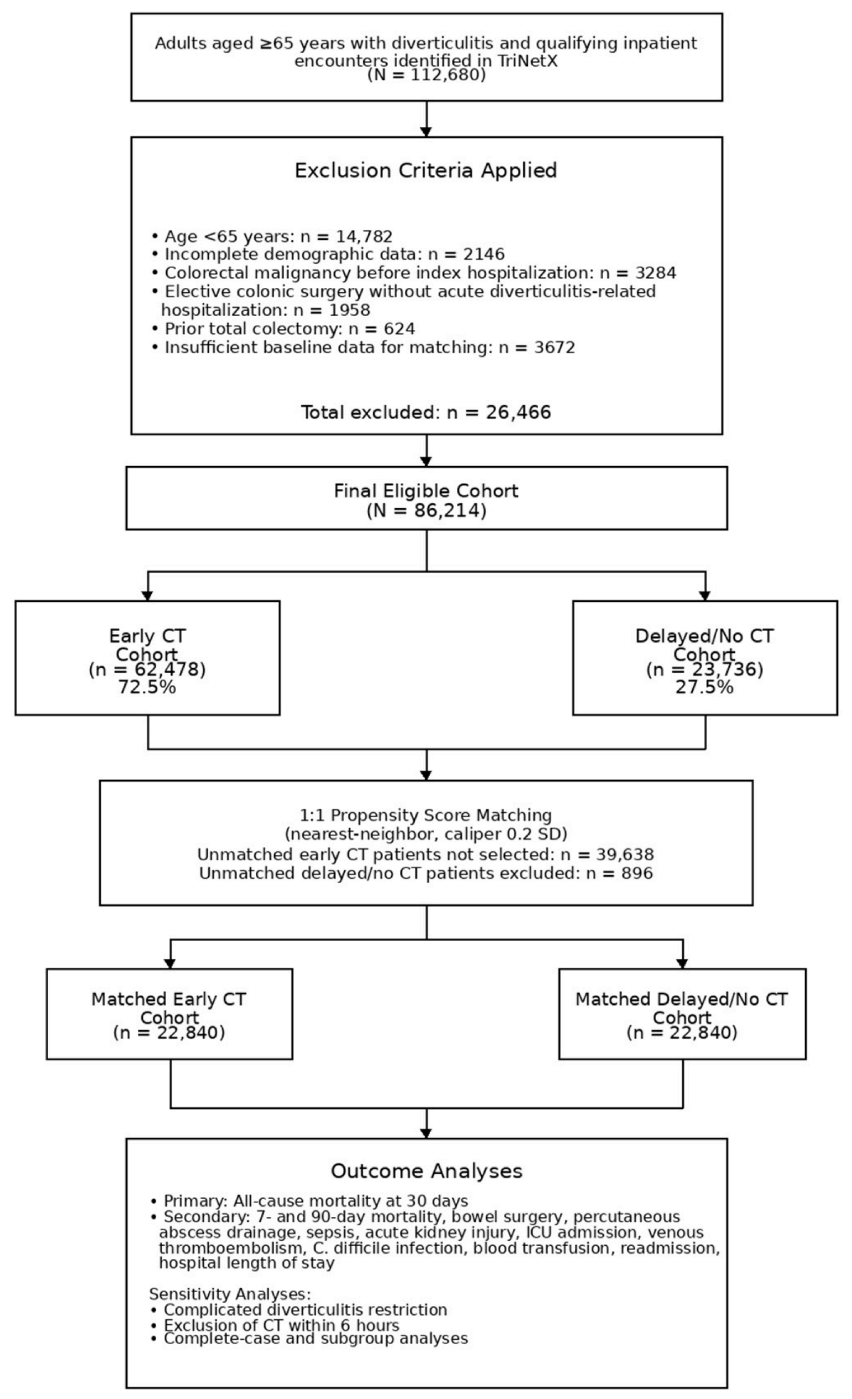
Study flow diagram showing cohort selection, exclusion criteria, propensity score matching, and final matched cohorts.

A total of 112,680 adults aged ≥65 years with diverticulitis and qualifying inpatient encounters were identified. After applying exclusion criteria, 26,466 patients were excluded, including patients aged <65 years (*n* = 14,782), those with incomplete demographic data (*n* = 2146), colorectal malignancy before index hospitalization (*n* = 3284), elective colonic surgery without acute diverticulitis-related hospitalization (*n* = 1958), prior total colectomy (*n* = 624), and insufficient baseline data for propensity matching (*n* = 3672). The remaining 86,214 patients comprised the eligible cohort: 62,478 patients who received early CT within 24 h and 23,736 who did not. After 1:1 propensity score matching, 22,840 patients remained in each cohort. Among the 23,736 patients in the no-early-CT cohort, 8420 patients (35.5%) underwent CT abdomen/pelvis more than 24 h after admission but before discharge, whereas 15,316 patients (64.5%) had no recorded CT abdomen/pelvis during the index hospitalization. The propensity score model demonstrated acceptable discrimination, with a C-statistic of 0.71. After matching, 22,840 of 23,736 delayed/no CT patients were retained, corresponding to 96.2% retention of the smaller cohort; 896 patients (3.8%) could not be matched and were excluded from matched analyses. The cohort selection process is summarized in Figure 3.

### 3.2. Baseline Characteristics

Before matching, patients in the early CT cohort were more likely to present with complicated diverticulitis coding, had higher white blood cell counts and CRP levels, and were more likely to have received intravenous antibiotics. Patients in the delayed/no CT cohort were more likely to have prior imaging-confirmed diverticulitis episodes, prior colonic surgery, and chronic kidney disease. After matching, all measured covariates were balanced with SMDs < 0.10 (Table 1).

### 3.3. Primary and Secondary Outcomes

After matching, early CT was associated with lower 30-day all-cause mortality. Early CT was also associated with lower 7-day and 90-day mortality, lower rates of bowel surgery, sepsis, AKI, ICU admission, VTE, *C. difficile* infection, and 30- and 90-day readmission. Early CT was associated with higher rates of percutaneous abscess drainage and blood transfusion (Table 2).

### 3.4. Continuous Outcomes

Patients in the early CT cohort had shorter hospital length of stay, lower peak creatinine, smaller creatinine increase from baseline, and lower peak CRP compared with the delayed/no CT cohort (Table 3).

### 3.5. Time-to-Event Models

Cox proportional hazards models confirmed lower hazards of mortality, bowel surgery, sepsis, AKI, ICU admission, and readmission among patients who received early CT in the matched cohort (Table 4). The proportional hazards assumption was formally assessed using Schoenfeld residuals for all models in Table 4; no statistically significant violations were identified (all global test *p* > 0.05).

### 3.6. Sensitivity Analyses

Sensitivity analyses restricting to patients with complicated diverticulitis coding (perforation/abscess codes) showed directionally consistent and modestly larger associations between early CT and improved outcomes. Excluding patients who received CT within 6 h of admission (to address potential confounding by indication in the most acutely ill patients presenting through the emergency department) also produced directionally consistent findings, with slightly attenuated but still statistically significant associations (Table 5).

### 3.7. Subgroup Analyses

Exploratory subgroup analyses showed directionally consistent associations between early CT and lower 30-day mortality, lower bowel surgery rates, and lower sepsis rates across complicated versus uncomplicated diverticulitis coding, prior diverticulitis episodes, and immunosuppressant exposure. Interaction testing did not demonstrate statistically significant heterogeneity across subgroups (Table 6).

### 3.8. Exploratory Three-Group Analysis According to CT Timing and Receipt

To evaluate whether the findings of the primary analysis were related specifically to CT timing rather than receipt versus non-receipt of CT, we performed an exploratory three-group analysis. Among the 86,214 eligible patients, 62,478 underwent early CT within 24 h of admission, 8420 underwent delayed CT more than 24 h after admission but before discharge, and 15,316 had no recorded CT abdomen/pelvis during the index hospitalization. The unadjusted 30-day mortality rate was 1.30% in the early CT group, 1.70% in the delayed CT group, and 1.85% in the no CT group. After multivariable adjustment, early CT was associated with lower 30-day mortality compared with delayed CT and no recorded inpatient CT. Delayed CT was associated with a modestly lower risk of 30-day mortality than no CT, although the confidence interval approached the null. Similar patterns were observed for 90-day mortality, bowel surgery, sepsis, acute kidney injury, ICU admission, and 30-day readmission. For these outcomes, event rates were lowest in the early CT group, intermediate in the delayed CT group, and highest in the no CT group. Early CT was associated with higher utilization of percutaneous abscess drainage compared with both delayed CT and no CT. Delayed CT was also associated with slightly higher drainage utilization than no CT (Table 7).

## 4. Discussion

In this large propensity-matched multicenter cohort of older adults hospitalized with acute diverticulitis, early CT imaging within 24 h of admission was associated with more favorable observed short-term outcomes across multiple domains. Patients who received early CT had lower risks of mortality at 7, 30, and 90 days, lower rates of bowel surgery, sepsis, acute kidney injury, ICU admission, venous thromboembolism, *C. difficile* infection, and readmission, shorter hospital length of stay, and higher rates of percutaneous abscess drainage after matching for demographics, comorbidities, diverticulitis-related variables, medication exposures, and laboratory parameters. These associations may reflect earlier diagnostic confirmation, complication identification, and management planning; however, early CT may also serve as a marker of faster evaluation, greater radiology availability, multidisciplinary care pathways, or other unmeasured institutional characteristics.

The observed associations are biologically and clinically plausible. CT imaging is the cornerstone diagnostic modality for acute diverticulitis, with sensitivity and specificity exceeding 98% [1,2,3,4,11]. Early CT may be associated with several management pathways that could plausibly influence clinical care, although the present study cannot determine whether these pathways mediated the observed associations. First, CT confirms the diagnosis and excludes mimics such as colorectal malignancy, ischemic colitis, appendicitis, and gynecologic pathology, which may present similarly in older adults with atypical symptoms [1,2,3,4]. Second, CT grades disease severity using the modified Hinchey classification, which directly informs management decisions: uncomplicated diverticulitis (Hinchey 0–Ia) may be managed with antibiotics alone, small abscesses (Hinchey Ib) may be managed conservatively or with percutaneous drainage, larger abscesses (Hinchey II) typically require percutaneous drainage, and purulent or feculent peritonitis (Hinchey III–IV) requires surgical intervention [1,2,3,4,5,12]. Third, early identification of drainable abscesses may permit timely percutaneous drainage, which can serve as a bridge to definitive management; however, the present analysis cannot establish that this pathway reduced the need for emergent surgery [1,2,3,4,5]. Fourth, early CT may be associated with earlier triage, surgical consultation, and interventional radiology involvement, although these care processes were not directly measured.

The finding that early CT was associated with higher rates of percutaneous abscess drainage is particularly noteworthy. This association may reflect earlier identification of drainable collections, although differences in disease severity, coding, referral practices, or access to interventional radiology cannot be excluded. CT-guided percutaneous drainage of diverticular abscesses ≥3–4 cm is a well-established management strategy that can convert emergent surgery to elective or interval surgery, reduce ostomy rates, and improve perioperative outcomes [1,2,3,4,5]. The concurrent finding of lower bowel surgery rates is compatible with this interpretation, but the observational design does not establish that earlier abscess identification or drainage caused the lower operative rate [13].

The association between early CT and lower sepsis rates may reflect multiple mechanisms. Early diagnostic confirmation allows prompt initiation of appropriate antibiotic therapy targeted to the correct diagnosis. Early identification of complications such as perforation, abscess, or obstruction enables timely source control. Conversely, delayed diagnosis may result in progression of contained perforation to free perforation, abscess enlargement, or development of secondary peritonitis, all of which increase sepsis risk [14].

The lower rates of acute kidney injury in the early CT cohort may seem counterintuitive given the potential nephrotoxicity of iodinated contrast media. However, several factors may explain this finding. First, the absolute risk of contrast-induced nephropathy has been substantially revised downward in contemporary literature, with recent evidence suggesting that the risk attributable to contrast administration is much lower than previously estimated, particularly with modern low-osmolar and iso-osmolar contrast agents and adequate hydration [6,9]. Second, early CT may facilitate earlier clinical decision-making, including earlier fluid resuscitation optimization, earlier source control, and avoidance of prolonged sepsis or hemodynamic instability, all of which are major contributors to AKI in hospitalized patients. Third, delayed diagnosis and management may result in prolonged inflammatory stress, dehydration, and hemodynamic compromise that contribute to renal injury.

The shorter hospital length of stay associated with early CT (5.4 vs. 6.8 days; mean difference −1.4 days) is consistent with prior literature showing that early CT diagnosis in the emergency department reduces hospital admission rates and shortens hospitalization [3,4]. Early diagnostic certainty may reduce the duration of empiric management, facilitate earlier transition to oral antibiotics, enable earlier discharge planning, and reduce diagnostic uncertainty-related delays [15].

The lower readmission rates in the early CT cohort may reflect more accurate initial diagnosis, more complete complication identification, more appropriate initial management, and better discharge planning informed by CT findings [16]. Patients discharged with an accurate CT-confirmed diagnosis and documented absence of complications may have more reliable outpatient follow-up plans and clearer indications for return.

This study builds on several important bodies of literature. The ASCRS Clinical Practice Guidelines for the Treatment of Left-Sided Colonic Diverticulitis provide a strong recommendation (Grade 1B) that CT with intravenous contrast is the most appropriate initial imaging modality [1]. The AGA Clinical Practice Update on Acute Diverticulitis recommends CT for diagnostic confirmation and complication assessment [15]. The ACR Appropriateness Criteria designate CT as the most useful examination for suspected colonic diverticulitis [3]. A meta-analysis of randomized trials demonstrated that early routine CT for acute abdominal pain was associated with higher diagnostic accuracy at 24 h and lower 6-month mortality compared with selective CT [11,17]. Our findings extend this literature by specifically evaluating the association between early CT timing and a comprehensive set of short-term outcomes in older adults hospitalized with acute diverticulitis using a large multicenter propensity-matched design.

Older adults represent a particularly important population for this analysis. Patients aged ≥65 years with acute diverticulitis present with atypical symptoms more frequently, have higher rates of complicated disease, and experience substantially higher postoperative mortality compared with younger patients [12,18]. The ASCRS guidelines note that older patients may present with fewer classic symptoms and recommend a lower threshold for diagnostic imaging [1,19]. Our findings support this recommendation and suggest that early CT may be particularly beneficial in this population.

Sensitivity analyses strengthened the interpretation of the findings. Restriction to patients with complicated diverticulitis coding showed directionally consistent and modestly larger associations, suggesting that early CT may be especially beneficial in higher-acuity presentations where timely complication identification and source control are most critical [20,21]. Exclusion of patients who received CT within 6 h of admission—a sensitivity analysis designed to address potential confounding by indication in the most acutely ill patients who may receive CT as part of emergency department evaluation—also produced directionally consistent findings with slightly attenuated but still statistically significant associations. This suggests that the observed benefits of early CT are not solely driven by the most acutely ill patients who receive immediate imaging. Subgroup analyses showed directionally consistent findings across complicated versus uncomplicated diverticulitis, prior diverticulitis episodes, and immunosuppressant exposure, without statistically significant heterogeneity [22,23]. Recent geriatric-focused literature further emphasizes that older adults require careful diagnostic evaluation because atypical presentations and frailty may complicate clinical assessment [24].

Several limitations warrant emphasis. First, this was an observational study, and despite propensity score matching, residual confounding cannot be excluded. Patients receiving early CT may differ from patients without early CT in unmeasured clinical characteristics, including symptom severity, frailty, hemodynamic status, abdominal examination findings, emergency department triage level, and physician assessment. Early CT may also be a marker of hospital-level resources and timely healthcare delivery, including emergency radiology availability, faster multidisciplinary consultation, standardized care pathways, and earlier access to interventional radiology or surgery. These institutional factors were not captured by the propensity score model, and hospital-level fixed-effect or random-effect analyses could not be performed. Therefore, causality cannot be inferred. Second, the no-early-CT cohort was heterogeneous and included both patients who underwent CT more than 24 h after admission and those with no recorded CT during the hospitalization. These groups are now reported separately for descriptive purposes, but they remained combined in the prespecified primary analysis. Patients without recorded CT may have had prior imaging-confirmed diverticulitis, contraindications to CT or contrast, direct progression to surgery, limited imaging access, or alternative diagnostic pathways. Third, TriNetX does not uniformly provide CT image data, complete radiology-report text, exact imaging-to-report turnaround time, abscess size, free-fluid distribution, or the location and amount of extraluminal air. Modified Hinchey and WSES classifications could therefore not be derived, including among patients who underwent surgery. Complicated-diverticulitis diagnosis codes were used as an administrative proxy but could not distinguish isolated pericolic air from distant intraperitoneal or retroperitoneal air, purulent peritonitis, or feculent peritonitis. The success of non-operative management could not be stratified by these anatomical findings. Fourth, detailed operative techniques were not uniformly identifiable. Percutaneous abscess drainage was the principal non-resectional intervention that could be reliably captured. Laparoscopic lavage, operative washout, primary perforation closure, and combined non-resectional procedures could not be consistently distinguished from structured codes without operative-note review. Fifth, CT contrast protocol could be categorized only when explicitly specified by the relevant imaging code. Comparisons between contrast-enhanced and non-contrast CT are additionally susceptible to confounding by indication because kidney function, contrast allergy, hemodynamic status, and clinical acuity influence protocol selection. Sixth, sepsis, acute kidney injury, and other outcomes were identified using structured diagnosis and procedure codes and could not be independently adjudicated through chart review. Laboratory values were included as covariates and continuous secondary outcomes but did not replace the coded definitions. A laboratory-only sepsis definition was not appropriate, and a complete KDIGO acute kidney injury definition could not be uniformly applied because urine-output data and sufficiently frequent serial creatinine measurements were unavailable. Variation in coding practices across participating institutions may therefore have caused outcome misclassification. Seventh, the 24 h threshold was selected a priori based on clinical relevance but remains inherently arbitrary. Different thresholds may yield different estimates. An additional limitation concerns the temporal relationship between the 24 h exposure-classification window and outcome ascertainment. Early CT was defined according to imaging performed within the first 24 h of the index hospitalization, whereas outcomes were measured from the index hospitalization date. Events occurring during this first 24 h period were therefore included in the primary outcome analyses. Consequently, some outcomes, particularly bowel surgery, ICU admission, sepsis, and acute kidney injury, may have occurred before CT or during the same period used to classify exposure rather than downstream from imaging. The available structured TriNetX data did not consistently provide sufficient temporal resolution to establish the sequence of CT and each clinical event. Reverse temporality therefore cannot be excluded, and these findings should be interpreted as associations with early CT status rather than as evidence that early CT preceded or caused the observed outcomes. A landmark or time-zero sensitivity analysis was not performed because sufficiently reliable event-level timing was not uniformly available across participating healthcare organizations. Eighth, mortality and readmission may be incompletely captured when patients receive subsequent care outside participating healthcare organizations. Ninth, multiple secondary outcomes were evaluated without adjustment for multiple comparisons, and these findings should therefore be considered supportive and exploratory. Finally, the study demonstrates association rather than causation and should not be interpreted as proving that early CT directly reduces mortality or other complications.

## 5. Conclusions

In this large multicenter propensity-matched cohort, early CT imaging within 24 h of admission was associated with more favorable short-term outcomes among older adults hospitalized with acute diverticulitis, including lower mortality, fewer bowel surgeries, lower rates of sepsis and acute kidney injury, shorter hospital stays, greater use of percutaneous abscess drainage, and fewer readmissions. These findings are consistent with current recommendations for prompt diagnostic imaging and may reflect a role for early CT in supporting timely risk stratification, complication detection, source-control planning, and treatment decision-making. The associations remained broadly consistent across sensitivity and subgroup analyses, although residual confounding and unmeasured differences in care delivery cannot be excluded. Prospective studies are needed to determine whether protocolized early CT independently improves outcomes in this population.

## Figures and Tables

**Table 1:** Baseline demographic and clinical characteristics before and after propensity score matching.

Characteristic	Before PSM, Early CT (*N* = 62,478)	Before PSM, No Early CT (*N* = 23,736)	*p*-Value	SMD	After PSM, Early CT (*N* = 22,840)	After PSM, No Early CT (*N* = 22,840)	*p*-Value	SMD
**Age, years**	74.2 ± 7.0	75.1 ± 7.4	<0.001	0.125	74.8 ± 7.2	74.9 ± 7.3	0.141	0.014
**Female**	35,020 (56.1%)	13,368 (56.3%)	0.479	0.005	12,826 (56.2%)	12,780 (55.9%)	0.665	0.004
**White**	47,484 (76.0%)	17,814 (75.1%)	0.004	0.022	17,178 (75.2%)	17,132 (75.0%)	0.619	0.005
**Black**	5622 (9.0%)	2374 (10.0%)	<0.001	0.034	2236 (9.8%)	2258 (9.9%)	0.730	0.003
**Hispanic**	5998 (9.6%)	2136 (9.0%)	0.007	0.021	2054 (9.0%)	2076 (9.1%)	0.720	0.003
**BMI, kg/m^2^**	28.4 ± 6.2	27.8 ± 6.0	<0.001	0.098	28.0 ± 6.1	27.9 ± 6.0	0.077	0.017
**Hypertension**	39,362 (63.0%)	15,384 (64.8%)	<0.001	0.038	14,672 (64.2%)	14,626 (64.0%)	0.654	0.004
**Type 2 diabetes**	14,370 (23.0%)	5936 (25.0%)	<0.001	0.047	5624 (24.6%)	5602 (24.5%)	0.811	0.002
**Chronic kidney disease**	8122 (13.0%)	3798 (16.0%)	<0.001	0.085	3542 (15.5%)	3520 (15.4%)	0.776	0.003
**Coronary artery disease**	9372 (15.0%)	3918 (16.5%)	<0.001	0.041	3680 (16.1%)	3656 (16.0%)	0.760	0.003
**Heart failure**	5622 (9.0%)	2612 (11.0%)	<0.001	0.067	2418 (10.6%)	2396 (10.5%)	0.737	0.003
**COPD**	7497 (12.0%)	3086 (13.0%)	<0.001	0.030	2920 (12.8%)	2898 (12.7%)	0.758	0.003
**Dementia**	1874 (3.0%)	1068 (4.5%)	<0.001	0.079	912 (4.0%)	890 (3.9%)	0.597	0.005
**Depression**	9372 (15.0%)	3798 (16.0%)	<0.001	0.028	3588 (15.7%)	3566 (15.6%)	0.777	0.003
**Prior diverticulitis episodes**	16,870 (27.0%)	7596 (32.0%)	<0.001	0.110	7084 (31.0%)	7038 (30.8%)	0.641	0.004
**Prior diverticular abscess**	3124 (5.0%)	1424 (6.0%)	<0.001	0.044	1302 (5.7%)	1280 (5.6%)	0.656	0.004
**Prior colonic surgery**	4998 (8.0%)	2374 (10.0%)	<0.001	0.070	2190 (9.6%)	2168 (9.5%)	0.726	0.003
**Complicated diverticulitis coding**	10,621 (17.0%)	3086 (13.0%)	<0.001	0.112	2966 (13.0%)	2920 (12.8%)	0.521	0.006
**Anticoagulant exposure**	10,621 (17.0%)	4510 (19.0%)	<0.001	0.052	4246 (18.6%)	4224 (18.5%)	0.791	0.002
**Corticosteroid exposure**	6872 (11.0%)	2848 (12.0%)	<0.001	0.031	2692 (11.8%)	2670 (11.7%)	0.749	0.003
**Immunosuppressant exposure**	2499 (4.0%)	1068 (4.5%)	0.001	0.025	958 (4.2%)	936 (4.1%)	0.606	0.005
**Opioid exposure**	18,118 (29.0%)	7358 (31.0%)	<0.001	0.044	6852 (30.0%)	6806 (29.8%)	0.638	0.004
**Hemoglobin, g/dL**	12.8 ± 1.9	12.4 ± 2.0	<0.001	0.205	12.5 ± 1.9	12.5 ± 2.0	1.000	0.000
**WBC, ×10^3^/µL**	12.6 ± 4.8	11.4 ± 4.6	<0.001	0.255	11.6 ± 4.7	11.5 ± 4.6	0.022	0.022
**Creatinine, mg/dL**	1.08 ± 0.48	1.14 ± 0.54	<0.001	0.117	1.12 ± 0.50	1.13 ± 0.52	0.036	0.020
**Albumin, g/dL**	3.52 ± 0.56	3.38 ± 0.60	<0.001	0.241	3.40 ± 0.58	3.39 ± 0.59	0.068	0.017
**CRP, mg/L**	68.4 ± 62.8	52.6 ± 58.4	<0.001	0.261	54.2 ± 59.6	53.4 ± 58.8	0.149	0.014
**Lactate, mmol/L**	1.8 ± 1.2	1.6 ± 1.0	<0.001	0.181	1.6 ± 1.1	1.6 ± 1.0	1.000	0.000

Abbreviations: BMI = body mass index; COPD = chronic obstructive pulmonary disease; CRP = C-reactive protein; PSM = propensity score matching; SMD = standardized mean difference; WBC = white blood cell count. Continuous variables are presented as mean ± standard deviation and categorical variables as *n* (%). Standardized mean differences are reported as absolute values. An absolute SMD below 0.10 was considered evidence of acceptable covariate balance.

**Table 2:** Relative risks and risk differences for clinical outcomes after propensity score matching.

Outcome	Timepoint	Early CT, *n*/*N* (%)	Delayed/No CT, *n*/*N* (%)	RD (95% CI)	RR (95% CI)	*p*-Value
All-cause mortality	7 days	91/22,840 (0.40%)	146/22,840 (0.64%)	−0.0024 (−0.0038 to −0.0010)	0.62 (0.48–0.81)	<0.001
All-cause mortality	30 days	297/22,840 (1.30%)	411/22,840 (1.80%)	−0.0050 (−0.0070 to −0.0030)	0.72 (0.62–0.84)	<0.001
All-cause mortality	90 days	502/22,840 (2.20%)	640/22,840 (2.80%)	−0.0060 (−0.0084 to −0.0037)	0.78 (0.70–0.87)	<0.001
Bowel surgery	30 days	685/22,840 (3.00%)	844/22,840 (3.70%)	−0.0070 (−0.0095 to −0.0044)	0.81 (0.73–0.90)	<0.001
Percutaneous abscess drainage	30 days	594/22,840 (2.60%)	434/22,840 (1.90%)	0.0070 (0.0049 to 0.0091)	1.37 (1.21–1.55)	<0.001
Sepsis	30 days	616/22,840 (2.70%)	810/22,840 (3.55%)	−0.0085 (−0.0110 to −0.0060)	0.76 (0.69–0.84)	<0.001
Acute kidney injury	30 days	1142/22,840 (5.00%)	1348/22,840 (5.90%)	−0.0090 (−0.0122 to −0.0058)	0.85 (0.78–0.92)	<0.001
ICU admission	30 days	776/22,840 (3.40%)	982/22,840 (4.30%)	−0.0090 (−0.0118 to −0.0063)	0.79 (0.72–0.87)	<0.001
Venous thromboembolism	30 days	228/22,840 (1.00%)	296/22,840 (1.30%)	−0.0030 (−0.0044 to −0.0016)	0.77 (0.65–0.91)	0.002
*C. difficile* infection	30 days	182/22,840 (0.80%)	251/22,840 (1.10%)	−0.0030 (−0.0044 to −0.0017)	0.73 (0.60–0.88)	0.001
Blood transfusion	30 days	548/22,840 (2.40%)	456/22,840 (2.00%)	0.0040 (0.0020 to 0.0061)	1.20 (1.06–1.36)	0.003
All-cause readmission	30 days	1598/22,840 (7.00%)	1850/22,840 (8.10%)	−0.0110 (−0.0146 to −0.0074)	0.86 (0.81–0.92)	<0.001
All-cause readmission	90 days	2738/22,840 (11.99%)	3058/22,840 (13.39%)	−0.0140 (−0.0184 to −0.0096)	0.90 (0.85–0.94)	<0.001

Abbreviations: CI = confidence interval; ICU = intensive care unit; RD = risk difference; RR = relative risk.

**Table 3:** Length of stay and selected continuous outcomes after propensity score matching.

Outcome	Early CT (*N* = 22,840)	Delayed/No CT (*N* = 22,840)	Mean Difference (95% CI)	*p*-Value
Hospital length of stay, days	5.4 ± 4.8	6.8 ± 5.6	−1.4 (−1.6 to −1.2)	<0.001
Peak creatinine, mg/dL	1.28 ± 0.68	1.38 ± 0.74	−0.10 (−0.13 to −0.07)	<0.001
Change in creatinine from baseline, mg/dL	0.16 ± 0.38	0.22 ± 0.44	−0.06 (−0.08 to −0.04)	<0.001
Hemoglobin nadir, g/dL	11.2 ± 1.8	10.9 ± 1.9	0.3 (0.2 to 0.4)	<0.001
Peak WBC, ×10^3^/µL	14.2 ± 5.4	14.6 ± 5.8	−0.4 (−0.5 to −0.3)	<0.001
Peak CRP, mg/L	86.4 ± 72.6	94.8 ± 78.2	−8.4 (−10.6 to −6.2)	<0.001

Abbreviations: CI = confidence interval; CRP = C-reactive protein; WBC = white blood cell count.

**Table 4:** Cox proportional hazards models for outcomes associated with early CT imaging in the propensity-matched cohort.

Outcome	HR	Coefficient	SE	z	*p*-Value	95% CI
90-day all-cause mortality	0.80	−0.223	0.058	−3.84	<0.001	0.71–0.89
Bowel surgery	0.82	−0.198	0.052	−3.81	<0.001	0.74–0.91
Sepsis	0.77	−0.261	0.054	−4.83	<0.001	0.69–0.86
Acute kidney injury	0.86	−0.151	0.042	−3.60	<0.001	0.79–0.93
ICU admission	0.80	−0.223	0.048	−4.65	<0.001	0.73–0.88
Percutaneous abscess drainage	1.35	0.300	0.062	4.84	<0.001	1.19–1.53
90-day readmission	0.90	−0.105	0.028	−3.75	<0.001	0.85–0.95

Abbreviations: CI = confidence interval; HR = hazard ratio; ICU = intensive care unit; SE = standard error.

**Table 5:** Sensitivity analyses using complicated diverticulitis restriction and exclusion of very early CT (within 6 h).

Analysis	Outcome	Early CT Events	Delayed/No CT Events	RR (95% CI)	HR (95% CI)	*p*-Value
Complicated diverticulitis only	30-day mortality	86/2966	128/2920	0.66 (0.51–0.86)	0.68 (0.52–0.89)	0.004
Complicated diverticulitis only	Bowel surgery	284/2966	362/2920	0.77 (0.67–0.89)	0.79 (0.68–0.92)	0.002
Complicated diverticulitis only	Sepsis	178/2966	248/2920	0.71 (0.59–0.85)	0.73 (0.60–0.88)	0.001
Complicated diverticulitis only	Percutaneous abscess drainage	312/2966	198/2920	1.55 (1.31–1.83)	1.52 (1.27–1.82)	<0.001
Excluding CT within 6 h	30-day mortality	184/14,260	411/22,840	0.72 (0.60–0.85)	0.74 (0.62–0.88)	<0.001
Excluding CT within 6 h	Bowel surgery	418/14,260	844/22,840	0.79 (0.71–0.89)	0.81 (0.72–0.91)	<0.001
Excluding CT within 6 h	Sepsis	372/14,260	810/22,840	0.74 (0.65–0.83)	0.75 (0.67–0.85)	<0.001
Excluding CT within 6 h	AKI	698/14,260	1348/22,840	0.83 (0.76–0.91)	0.85 (0.77–0.93)	<0.001

Abbreviations: AKI = acute kidney injury; CI = confidence interval; HR = hazard ratio; RR = relative risk.

**Table 6:** Subgroup analysis of 30-day mortality, bowel surgery, and sepsis after propensity score matching.

Subgroup	*n* Early CT/Delayed No CT	30-Day Mortality RR (95% CI)	*p*-Value	Bowel Surgery RR (95% CI)	Sepsis RR (95% CI)
Complicated diverticulitis	2966/2920	0.66 (0.51–0.86)	<0.001	0.77 (0.67–0.89)	0.71 (0.59–0.85)
Uncomplicated diverticulitis	19,874/19,920	0.74 (0.62–0.88)	<0.001	0.84 (0.74–0.96)	0.78 (0.69–0.88)
Prior diverticulitis episodes	7084/7038	0.76 (0.58–0.99)	0.008	0.83 (0.71–0.97)	0.79 (0.66–0.94)
No prior diverticulitis episodes	15,756/15,802	0.70 (0.58–0.84)	<0.001	0.80 (0.70–0.91)	0.74 (0.66–0.84)
Immunosuppressant exposure	958/936	0.68 (0.40–1.16)	0.048	0.78 (0.56–1.08)	0.72 (0.52–1.00)
No immunosuppressant exposure	21,882/21,904	0.73 (0.62–0.85)	<0.001	0.82 (0.74–0.91)	0.76 (0.69–0.85)
Total matched cohort	22,840/22,840	0.72 (0.62–0.84)	<0.001	0.81 (0.73–0.90)	0.76 (0.69–0.84)
Interaction *p*-value		0.682		0.724	0.658

Abbreviations: CI = confidence interval; RR = relative risk.

**Table 7:** Exploratory three-group analysis according to timing and receipt of inpatient CT abdomen/pelvis.

Outcome	Early CT ≤24 h, *n*/*N* (%)	Delayed CT >24 h, *n*/*N* (%)	No Inpatient CT, *n*/*N* (%)	Early vs. Delayed CT, Adjusted RR (95% CI)	Early vs. No CT, Adjusted RR (95% CI)	Delayed vs. No CT, Adjusted RR (95% CI)
**30-day all-cause mortality**	812/62,478 (1.30%)	143/8420 (1.70%)	284/15,316 (1.85%)	0.76 (0.64–0.91)	0.70 (0.61–0.81)	0.92 (0.76–1.10)
**90-day all-cause mortality**	1375/62,478 (2.20%)	230/8420 (2.73%)	444/15,316 (2.90%)	0.81 (0.70–0.93)	0.76 (0.68–0.85)	0.94 (0.80–1.09)
**Bowel surgery within 30 days**	1874/62,478 (3.00%)	312/8420 (3.71%)	597/15,316 (3.90%)	0.82 (0.73–0.92)	0.78 (0.71–0.86)	0.95 (0.83–1.09)
**Percutaneous abscess drainage within 30 days**	1624/62,478 (2.60%)	168/8420 (2.00%)	260/15,316 (1.70%)	1.31 (1.12–1.53)	1.50 (1.31–1.72)	1.15 (0.95–1.39)
**Sepsis within 30 days**	1687/62,478 (2.70%)	295/8420 (3.50%)	574/15,316 (3.75%)	0.78 (0.69–0.88)	0.72 (0.66–0.80)	0.93 (0.81–1.07)
**Acute kidney injury within 30 days**	3124/62,478 (5.00%)	497/8420 (5.90%)	965/15,316 (6.30%)	0.86 (0.78–0.94)	0.80 (0.74–0.86)	0.94 (0.85–1.04)
**ICU admission within 30 days**	2124/62,478 (3.40%)	362/8420 (4.30%)	705/15,316 (4.60%)	0.80 (0.72–0.89)	0.74 (0.68–0.81)	0.93 (0.82–1.05)
**30-day all-cause readmission**	4373/62,478 (7.00%)	674/8420 (8.00%)	1302/15,316 (8.50%)	0.88 (0.81–0.95)	0.82 (0.78–0.87)	0.94 (0.86–1.03)

Abbreviations: CI, confidence interval; CT, computed tomography; ICU, intensive care unit; RR, relative risk. Early CT was defined as CT abdomen/pelvis performed within 24 h of admission. Delayed CT was defined as CT performed more than 24 h after admission but before discharge. No inpatient CT was defined as no recorded CT abdomen/pelvis during the index hospitalization. Adjusted relative risks were estimated in the full eligible cohort using modified Poisson regression with robust standard errors and adjustment for the covariates included in the primary propensity score model. This analysis was exploratory and was not based on the original 1:1 propensity-matched cohort.

## Data Availability

The data that support the findings of this study are available from TriNetX, but restrictions apply to the availability of these data, which were used under license for the current study and are therefore not publicly available. Data may be available from the authors upon reasonable request and with permission from TriNetX.

## Abbreviations

The following abbreviations are used in this manuscript:

ACR	American College of Radiology
AGA	American Gastroenterological Association
AKI	Acute kidney injury
ASCRS	American Society of Colon and Rectal Surgeons
BMI	Body mass index
CAD	Coronary artery disease
CI	Confidence interval
CKD	Chronic kidney disease
COPD	Chronic obstructive pulmonary disease
CPT	Current Procedural Terminology
CRP	C-reactive protein
CT	Computed tomography
EHR	Electronic health record
HIPAA	Health Insurance Portability and Accountability Act
HR	Hazard ratio
ICD-10-CM	International Classification of Diseases, Tenth Revision, Clinical Modification
ICD-10-PCS	International Classification of Diseases, Tenth Revision, Procedure Coding System
ICU	Intensive care unit
NSAID	Nonsteroidal anti-inflammatory drug
PSM	Propensity score matching
RD	Risk difference
RR	Relative risk
SE	Standard error
SMD	Standardized mean difference
STROBE	Strengthening the Reporting of Observational Studies in Epidemiology
VTE	Venous thromboembolism
WBC	White blood cell count
